# Familial cluster of cutaneous anthrax in North Khorasan, Iran: A case series and public health reflection

**DOI:** 10.1016/j.idcr.2025.e02444

**Published:** 2025-11-30

**Authors:** Mahnaz Arian, Syed Mohammad Naqvi, Narges Niazirad, Amir Javadi Torshizi, Azin Mohammadi, Kiana Ketabi

**Affiliations:** a Department of Infectious Diseases and Tropical Medicine, Faculty of Medicine, Mashhad University of Medical Sciences, Mashhad, Iran; b Student Research Committee, Mashhad University of Medical Sciences, Mashhad, Iran

**Keywords:** Anthrax, Bacillus anthracis, Cutaneous, Iran, Case series, Zoonosis

## Abstract

**Background:**

Cutaneous anthrax remains a persistent zoonotic threat in endemic agricultural regions. Iran, particularly North Khorasan Province, continues to report sporadic cases, especially among individuals involved in livestock processing.

**Case presentations:**

We describe a familial cluster of three cases of cutaneous anthrax following exposure to contaminated sheep meat in a rural village. All patients presented with characteristic black eschars on the hands and received initial inappropriate antibiotic treatment with cephalexin. Microbiological confirmation of *Bacillus anthracis* was achieved through smear and swab cultures. Subsequent treatment with ciprofloxacin led to full recovery in each case.

**Conclusion:**

These cases highlight the importance of accurate and timely diagnosis, the risks associated with the empirical use of ineffective antibiotics, and the need for clinician awareness in endemic settings. Public health responses should incorporate veterinary control measures, public education, and clinician training to prevent future cases.

## Introduction

Anthrax, an acute zoonotic infectious disease, is caused by *Bacillus anthracis*, a Gram-positive, spore-forming bacterium. Its capacity to form highly resistant endospores is fundamental to its persistence in the environment, contributing to the endemicity of the disease in many agricultural regions and posing a continuous challenge for control and eradication efforts [1]. Anthrax remains prevalent in areas of Africa, Asia, and the Middle East. Cutaneous anthrax accounts for over 95 % of human cases worldwide and is often linked to occupational exposure, with a notable male preponderance [2].

Cutaneous anthrax typically manifests following the entry of *Bacillus anthracis* spores into the skin via minor abrasions or wounds, acquired during the handling of infected animals or contaminated materials [3]. The local germination of spores and subsequent bacterial multiplication lead to toxin production, resulting in a characteristic clinical progression: an initially pruritic papule evolves into a vesicle, which then ulcerates to form a typically painless, necrotic black eschar surrounded by non-pitting edema [1]. While the painless nature of the lesion is a key diagnostic indicator, it may paradoxically contribute to delays in patients seeking medical care, allowing for disease progression.

If untreated, cutaneous anthrax can lead to systemic dissemination and carries a mortality rate of up to 20 %; however, with timely and appropriate antibiotic therapy, the prognosis is generally excellent [1]. Diagnosis relies on a combination of clinical suspicion, guided by epidemiological context (endemic area) and the presence of the characteristic eschar, with laboratory confirmation through methods such as Gram stain and culture of specimens from the lesion [1]. Prompt administration of effective antibiotics, such as fluoroquinolones or doxycycline, is critical for successful treatment [4], [5].

In Iran, the persistence of traditional livestock farming and home slaughter practices creates an ongoing risk for such zoonotic transmissions, motivating this report on three cases of cutaneous anthrax identified in North Khorasan Province, Iran [6]. These cases, arising from a common exposure to infected sheep meat, serve to illustrate the ongoing public health challenges posed by anthrax in such regions. We aim to highlight the importance of early and accurate diagnosis, the potential pitfalls associated with empirical antibiotic treatments that do not align with *Bacillus anthracis* susceptibility patterns, the effectiveness of guideline-based antimicrobial therapy, and stress the need for ongoing vigilance and coordinated control strategies. Reporting these cases with the initial clinical mismanagement provides a valuable learning opportunity for healthcare providers in similar settings, reinforcing best practices and heightening awareness of common diagnostic and therapeutic considerations.

## Case presentations

Three cases of cutaneous anthrax were identified in a village in North Khorasan Province, Iran, following exposure to infected sheep meat.

### Case 1

A 50-year-old woman presented with a superficial skin wound on her left index finger, sustained while cutting contaminated meat. One-week post-injury, her lesion developed swelling and erythema. Initial management with cefalexin (500 mg QID) and gentamicin (80 mg once daily) over three days failed to produce clinical improvement, and the swelling progressed into a characteristic black eschar. Given the epidemiological context and poor response, an infectious disease specialist was consulted. Smear and swab specimens from the lesion tested positive for *Bacillus anthracis*. Antibiotic therapy was adjusted to ciprofloxacin (500 mg BID) for 14 days. Follow-up demonstrated marked clinical improvement, including reduced edema and resolution of the eschar.

### Case 2

A 60-year-old male, husband of Case 1, also involved in preparing the infected meat, presented with a wound on his right thumb, covered by approximately a two cm black crust. Physical examination revealed ipsilateral axillary lymphadenopathy; all other findings were unremarkable. The patient had also been prescribed cefalexin (500 mg QID), which did not lead to any improvement. Wound swab confirmed the presence of *Bacillus anthracis*. He was subsequently treated with ciprofloxacin (500 mg BID) for 14 days, resulting in clinical improvement.

### Case 3

A 30-year-old woman, a family member involved in meat processing and cooking, exhibited a three cm ulcerative lesion on her right index finger, with a black crust and mild edema. Initially, she received cefalexin (500 mg QID) and gentamicin (160 mg once daily) for three days under the presumptive diagnosis of an infected wound. Due to a lack of clinical progress and similar exposure history, she was referred to an infectious disease specialist. Smear microscopy confirmed *B. anthracis*. Following a 14-day course of ciprofloxacin (500 mg BID), her wound showed signs of healing.

Please see Table 1 for the summary of cases.

**Table 1:** Clinical characteristics, management, and outcomes.

Characteristic	Case 1	Case 2	Case 3
**Age/Sex**	50 y/Female	60 y/Male	30 y/Woman
**Relation**	Wife of Case 2	Husband of Case 1	Family Member
**Exposure**	Cutting meat/Handling meat	Cutting meat/Handling meat	Meat processing/cooking
**Lesion Site**	Left Index Finger	Right Thumb	Right Index Finger
**Lesion Morphology**	Swelling, erythema, black eschar	Ulcerative lesion (2 cm) with black crust	Ulcerative lesion (3 cm) with black crust
**Systemic Signs**	None documented	Ipsilateral axillary lymphadenopathy	None documented
**Initial (Ineffective) Rx**	Cefalexin + Gentamicin	Cefalexin	Cefalexin + Gentamicin
**Definitive Treatment**	Ciprofloxacin (500 mg BID, 14 days)	Ciprofloxacin (500 mg BID, 14 days)	Ciprofloxacin (500 mg BID, 14 days)
**Outcome**	Full recovery & resolution of eschar	Full recovery & resolution of eschar	Full recovery & resolution of eschar

**Fig. 1:**
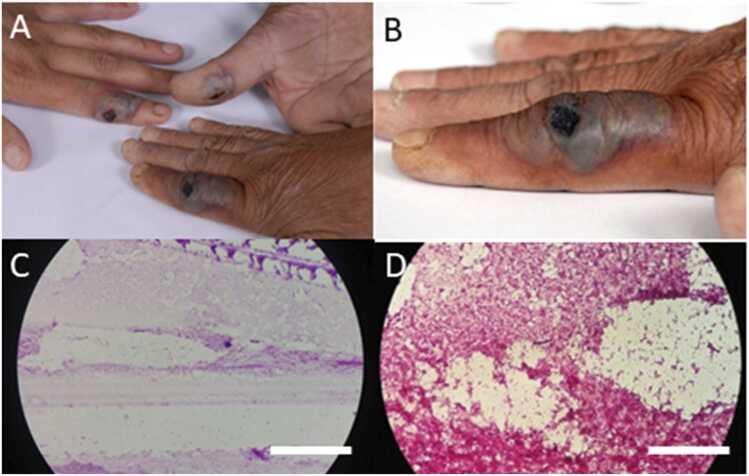
(A) Cutaneous skin lesions present on the hands of Patients 1, 2, and 3. (B) Cutaneous skin lesions present on the hand of Patient 2. (C)*Giemsa staining of the smear from Patient 2’s lesion. (D)*Gram staining of the smear from Patient 2’s lesion demonstrating a Gram-positive bacillus. *Scale bar = 5–20 μm.

In all three cases, the clinical timeline followed the characteristic progression. The lesions initially appeared as pruritic papules, which progressed to vesicles, eventually rupturing to form the painless, necrotic eschar observed on presentation (Fig. 1). All specimens for microbiological analysis were obtained following standard diagnostic protocols. Sterile swabs were used to collect fluid from beneath the margin of the eschar or from vesicular fluid to maximize the recovery of *Bacillus anthracis*. These samples were immediately sent for Gram staining and culture.

## Discussion

### Contextualization of the case series within Iranian epidemiology

Anthrax remains a significant, albeit sporadic, public health concern in Iran, particularly in regions with substantial livestock and agricultural activities. Iran reported the first recorded cases of anthrax in cattle in 1905 [7]. The primary mode of transmission identified in these three patients was direct contact with infected sheep meat during processing. A review of anthrax cases in Iran from 2000 to 2016 found that cutaneous anthrax constituted 56.6 % of human cases, frequently associated with exposure to animals or their products, including meat [8]. More recent reports, such as a 2022 outbreak in Kashan, also implicated contact with infected meat as the source of infection [9].

Northeast Iran, specifically the Khorasan region, has a documented history of anthrax endemicity. 28 cutaneous cases were recorded in Esfarayen, North Khorasan, in 2011, and veterinary surveillance in Khorasan Razavi province identified sporadic cases in sheep between 2013 and 2015 [8]. While cutaneous forms are the most common, severe presentations have also been noted in the region, including a fatal case of gastrointestinal anthrax in Bojnurd, North Khorasan, in 2015 following the consumption of infected goat meat [10]. This data confirms that the cases presented here are part of a persistent local zoonotic burden rather than isolated anomalies

The familial clustering observed, with two spouses and another family member involved in meat preparation, all contracting the disease, strongly points to a common-source exposure from a single infected animal. This underscores the elevated risk associated with handling and processing meat from animals that were slaughtered outside of regulated abattoirs, highlighting a critical point for public health intervention aimed at ensuring meat safety. North Khorasan Province fits the profile of an area where transmission of *Bacillus anthracis* is an ongoing risk, consistent with the occupational and environmental exposure patterns described in broader Iranian epidemiological studies.

### Diagnostic insights and challenges highlighted by the cases

The diagnostic pathway in these cases illustrates both the classic clinical signs and a delay in initial diagnosis. The development of a characteristic black eschar following a skin injury sustained during meat handling should, in an endemic area, immediately raise suspicion of cutaneous anthrax [1]. Confirmation in all three patients was achieved through smear microscopy and culture of *Bacillus anthracis* from lesion swabs, methods that remain fundamental in resource-limited settings [11]. The necessity for consultation with an infectious disease specialist after initial empirical treatment had failed suggests a potential gap in the awareness or diagnostic acumen for anthrax among primary healthcare providers in the region. This points towards a need for targeted educational initiatives for primary care physicians who are often the first point of contact for such patients.

While conventional microbiology was successful here, it is important to recognize that prior antibiotic administration can reduce the sensitivity of culture and Gram stain [11]. Timely and appropriate specimen collection, before the administration of effective antibiotics, is crucial for maximizing diagnostic yield. In situations with strong clinical suspicion but negative microbiological findings, more sensitive molecular diagnostic techniques, such as polymerase chain reaction (PCR), should be considered if available, as they can detect non-viable organisms or small quantities of bacterial DNA [11].

### Therapeutic management: lessons from initial pitfalls and subsequent success

An important aspect here was the initial antibiotic mismanagement. *Bacillus anthracis* is intrinsically resistant to many beta-lactam antibiotics, including cephalosporins, due to the production of beta-lactamases [12]. The empirical use of cefalexin (500 mg QID) in all three patients represents an inappropriate therapeutic choice and predictably resulted in a lack of clinical improvement. The addition of gentamicin, an aminoglycoside, was a second inappropriate choice to control the infection [12]. Only after starting ciprofloxacin (500 mg BID for 14 days) was there a marked clinical improvement and resolution. This outcome strongly validates current treatment guidelines from the U.S. Centers for Disease Control and Prevention (CDC) and Médecins Sans Frontières (MSF), which recommend fluoroquinolones (like ciprofloxacin) or doxycycline as first-line agents for uncomplicated cutaneous anthrax [4], [5], [12]. The 14-day duration of ciprofloxacin was appropriate, given the initial treatment failure and the nature of the lesions (e.g., a three cm ulcer in Case 3).

### Public health implications and recommendations

These cases serve as a stark reminder that cutaneous anthrax remains an ongoing public health challenge. The direct link to contaminated meat highlights the critical intersection of animal and human health, mandating a robust approach to control and prevention. Effective strategies must integrate veterinary and human health sectors. Specific risk behaviors include the handling of carcasses without personal protective equipment (PPE) and the manipulation of animal hides or wool, which are well-documented high-risk activities for cutaneous anthrax transmission.

Key recommendations include:

**Strengthening Public Health Measures:** This involves enhancing livestock vaccination programs against anthrax, which is a cornerstone of prevention [13]. Improved surveillance for anthrax in animal populations, coupled with strict enforcement of regulations for safe carcass disposal (e.g., incineration or deep burial to prevent spore dissemination) and abattoir hygiene, is essential to prevent contaminated animal products from entering the human food chain [13].

**Enhancing Human Health Surveillance and Education:** Continuous surveillance of human anthrax cases is necessary to monitor trends and detect outbreaks promptly. In rural settings, such as our case, home slaughter prevents veterinary inspection, significantly increasing exposure risk; therefore, public education can play an important role here. These campaigns should be targeted at at-risk populations, including farmers, butchers, veterinarians, and individuals involved in home slaughter and meat processing. Education should focus on transmission routes, the risks associated with handling or consuming meat, and the early signs of anthrax infection [8].

**Improving Clinical Capacity and Antimicrobial Stewardship:** There is a clear need for ongoing medical education for healthcare providers in endemic regions. This education should aim to improve early clinical suspicion of anthrax, enhance diagnostic capabilities (differentiating it from more common skin infections), and ensure adherence to current treatment guidelines. Promoting prompt consultation with infectious disease specialists for suspected cases or when initial treatments fail can improve patient outcomes. The misuse of antibiotics, as seen here, not only fails the individual patient but also contributes to broader antimicrobial resistance pressures if such practices are common for various undiagnosed infections.

The occurrence of these "simple" cutaneous anthrax cases should be viewed as sentinel events, indicating the continued circulation of *Bacillus anthracis* in the local animal population. This implies a persistent risk for more severe forms of anthrax, such as gastrointestinal or inhalational disease, should exposure circumstances differ (e.g., ingestion of heavily contaminated meat or aerosolization of spores). The socio-economic impact on families and communities, particularly those reliant on livestock, from both the disease itself and the fear it engenders, also warrants consideration in public health planning.

## Conclusion

This case series from North Khorasan Province underscores that cutaneous anthrax, transmitted through contact with contaminated meat, continues to pose a public health threat to this region. The cases highlight the critical importance of maintaining a high index of clinical suspicion among healthcare providers, especially when presented with characteristic lesions in individuals with relevant exposure histories. Accurate and timely diagnosis, coupled with adherence to guideline-recommended antibiotic therapy, especially avoiding empiric therapies ineffective for anthrax and utilizing appropriate agents, is paramount for successful patient outcomes. These experiences reaffirm the ongoing need for integrated health strategies, encompassing robust veterinary control measures, targeted public education, and continuous professional development for clinicians, to effectively mitigate the risks associated with anthrax and protect public health.

## CRediT authorship contribution statement

**Amir Javadi Torshizi:** Writing – review & editing, Data curation. **Kiana Ketabi:** Writing – review & editing, Visualization, Validation, Project administration. **Azin Mohammadi:** Writing – review & editing, Data curation. **Mahnaz Arian:** Writing – review & editing, Supervision, Project administration, Conceptualization. **Narges Niazirad:** Writing – review & editing, Investigation, Data curation. **Syed Mohammad Naqvi:** Writing – review & editing, Writing – original draft, Validation.

## Ethical approval

Written informed consent was obtained from all three patients for the publication of this report, including all accompanying images.

## Consent

Written informed consent was obtained from all three patients for the publication of this report, including all accompanying images.

## Declaration of Generative AI and AI-assisted technologies in the writing process

In the development and composition of this document, generative AI was not utilized beyond the scope of fundamental grammar, spelling, and reference verification tools.

## Funding

None.

## Patient Perspective

All three patients expressed gratitude for the medical team’s efforts in managing their infection and ensuring a successful outcome.

## Declaration of Competing Interest

The authors declare that they have no known competing financial interests or personal relationships that could have appeared to influence the work reported in this paper.
